# Interim pressure garment therapy (4–6 mmHg) and its effect on donor site healing in burn patients: study protocol for a randomised controlled trial

**DOI:** 10.1186/s13063-016-1329-x

**Published:** 2016-04-26

**Authors:** Michelle L. Donovan, Michael J. Muller, Claire Simpson, Michael Rudd, Jennifer Paratz

**Affiliations:** Occupational Therapy Department, Royal Brisbane and Women’s Hospital, Level 2, Dr James Mayne Building, Herston, QLD 4029 Australia; Burns, Trauma & Critical Care Research Centre, School of Medicine, University of Queensland, Herston, QLD 4029 Australia; Professor Stuart Pegg Adult Burns Centre, Royal Brisbane & Women’s Hospital, Level 4, Dr James Mayne Building, Herston, QLD 4029 Australia; School of Allied Health Sciences, Griffith University, Gold Coast Campus, Southport, QLD 4222 Australia

**Keywords:** Interim compression garment, Donor site, Burns, Occupational therapy

## Abstract

**Background:**

Pressure garment therapy (PGT) is well accepted and commonly used by clinicians in the treatment of burns scars and grafts. The medium to high pressures (24–40 mmHg) in these garments can support scar minimisation, and evidence is well documented for this particular application. However, PGT specifically for burn donor sites, of which a sequela is also scarring, is not well documented. This study protocol investigates the impact of a low pressure (4–6 mmHg) interim garment on donor site healing and scarring. With a primary purpose of holding donor dressings in place, the application of the interim pressure garment (IPG) appears to have been twofold. IPGs for donor sites have involved inconsistent application with a focus on securing wound dressing rather than scar management. However, anecdotal and observational evidence suggests that IPGs also make a difference to some patient’s scar outcomes for donor sites. This study protocol outlines a randomised controlled trial designed to test the effectiveness of this treatment on reducing scarring to burn donor sites.

**Methods/design:**

This study is a single-centre, single (assessor)-blinded, randomised control trial in patients with burns donor sites to their thighs. Patients will be randomly allocated to a control group (with no compression to donor sites) or to an experimental group (with compression to donor sites) as the comparative treatment. Groups will be compared at baseline regarding the important prognostic indicators: donor site location, depth, size, age, and time since graft (5 days). The IPG treatment will be administered post-operatively (on day 5). Follow-up assessments and garment replacement will be undertaken fortnightly for a period of 2 months.

**Discussion:**

This study focuses on a unique area of burns scar management using a low-pressure tubular support garment for the reduction of donor site scars. Such therapy specifically for donor scar management is poorly represented in the literature. This study was designed to test a potentially cost-effective scar prevention for patients with donor sites to the thigh. No known studies of this nature have been carried out to date, and there is a need for rigorous clinical evidence for low-pressure support garments for donor site scar minimisation.

**Trial registration:**

Australian New Zealand Clinical Trials Registry identifier ACTRN12610000127000. Registered 8 Mar 2010.

## Background

Thickened erythematous scars can reduce physical function and create a distorted physical appearance for a burn-injured patient [1–3]. When a burn penetrates deeply enough into the dermis of the skin, surgery is often required to assist healing. The surgery involves removing the burned or dead skin (debriding) and replacing it with a thin section of unburned healthy skin (donor skin), known as a split-thickness skin graft (SSG). This donor skin is surgically removed, commonly from the patient’s own thigh area, using a dermatome at a measure of 8/1000 inch to 12/1000 inch (depending on the thickness required to heal the burn defect) and includes the epidermis and the upper third of the dermis [4, 5]. The tissue removed for a SSG is comparable in depth to a partial thickness skin loss [5]. The remaining exposed area of dermis is referred to as the *donor site*. The donor skin is then placed over the original debrided burn wound area to assist in healing. There are sufficient residual epidermal cells in the remaining dermis to allow re-epithelisation to occur over an approximately 14-day period [4]. The donor site wounds discussed in this paper are routinely dressed with ALGISITE, ACTICOAT, MELOLIN, and HYPAFIX (all from Smith & Nephew, Andover, MA, USA) and heavy crepe bandage immediately post-operatively until wound review day 3 or 5. After this time, the donor site is then dressed with soft white paraffin (Kenkay Pharmaceuticals, Smeaton Grange, NSW, Australia), Xeroform (DeRoyal, Powell, TN, USA), and HYPAFIX or heavy crepe bandage until the wound is considered healed (no raw, open or oozing epithelium). Interim pressure garments (IPGs) for donor sites are routinely provided to inpatients at the Professor Pegg Adult Burns Centre, as early as day 1 post-operatively with a focus on securing wound dressings rather than scar management.

Despite the donor site depth being extremely thin, patients often describe this new wound to be like ‘bad gravel rash’ and report worse pain in this wound than at the original burn or graft site. Pain associated with burn surgery has been shown to delay patients’ return to work and daily functional activity, with donor sites consistently identified as a potential source of concern for burn patients [1–3, 6–8]. Protection and support of the remaining epidermal and dermal elements of this surgical wound (donor site) are required to assist effective tissue healing with minimal scarring [4, 5, 9–12].

A review of the literature published over the past decade demonstrates the advances made in minimising scar formation [13–15]. Compression garment therapy has been used since the early 1970s to aid healing of burns scars and graft sites [9, 11, 16–20]. It is believed that this is accomplished primarily by applying pressure to the healing burn wound with a medium- to high-pressure garment. The definition of medium to high pressure for burn garments has been reported in the literature to be between 20 mmHg and 40 mmHg [17, 21]. However, pressures as low as 15 mmHg have also been reported as being effective [15, 17, 22].

Pressure garments appear to enhance the scar maturation process following epithelisation and, if worn for a minimum of 20–23 h/day, also appear to inhibit the development of hypertrophic or abnormal scar tissue. Although the exact mechanism of scar reduction remains largely unknown [23], it is assumed to be due to the garment gently restricting blood flow to the scar surface area and reorganise collagen fibres to resemble that of normal skin through a constant external pressure to the skin surface [9, 10]. The reduction of capillary filtration into the wound bed has also been reported to assist in the reduction of pain for these patients [13, 24]. It is thought that the increased blood rush caused by gravity when standing, and particularly when the patient is mobilising, stretches the fragile surface of the donor site wound, resulting in pain [1–3, 6–8].

The pressure garment is positioned over any dressings that may be needed in the early stage of recovery and is worn until the burn scar has matured or become pale in colour, resembling normal skin. This process of scar maturation is reported to take 18–24 months to complete, but it can take longer in some cases [6, 8, 11, 19, 20]. Although it is an accepted intervention in reducing scar formation following a burn, the effectiveness of pressure garment therapy has not been scientifically assessed on donor site scars [6, 8, 9, 11, 16–21, 25, 26].

**Fig. 1:**
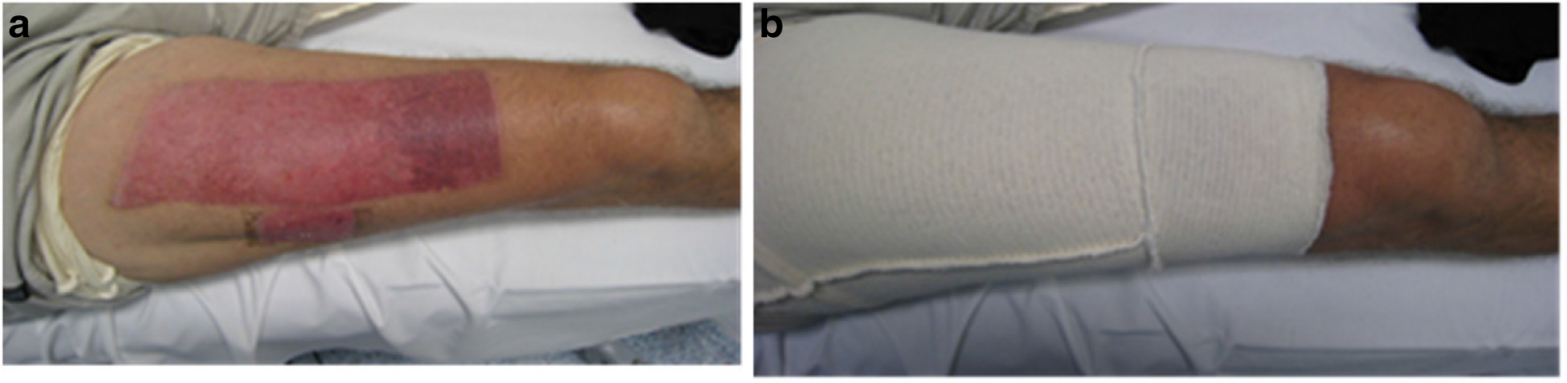
**a**. shows a healed donor scar located on the anterior thigh, that had application of IPG and the hypervascular distal donor that did not. **b**. The garment was extended to support the hypervascular distal donor

As an alternative, the gentler ‘interim’ garment (termed an *interim pressure garment* for the purpose of this study) can be applied post-operatively around days 3–5 before donning the firmer compression garment. These interim garments are made from a tubular elastic material (similar to Lycra® bicycle pants) (Fig. 1a) and apply a gentle supportive pressure over the thigh and donor site areas [7, 27].

Through the course of clinical practice, it has been observed that IPG appears to reduce donor site erythema and reported pain in some patients. Figure 1a shows a healed donor scar located on the anterior thigh, that had application of IPG and the hypervascular distal donor that did not. The garment was extended to support the hypervascular distal donor (Fig. 1b). However, the use of IPG in the treatment of burns donor sites has involved inconsistent application with a focus on wound dressing rather than scar management, and the amount of pressure (4–6 mmHg) that the garments applied was previously undefined. The current evidence to suggest that IPG may improve scar outcome in donor sites is therefore anecdotal and observational.

The levels of evidence outlining risks and benefits of pressure garment therapy have varied across the literature over the past 45 years. More recently, pressure garments (25–40 mmHg) have been shown to be effective for use in hypertrophic burns scars; however a paucity of evidence exists, along with inconsistent findings regarding the minimisation of erythema for such scars. These recommendations are reported as levels I and II evidence [7, 17, 21]. Despite this, pressure garment therapy is a well-known and well-used treatment by clinicians in reducing scar formation following a burn or graft; however, its effectiveness has not been reported on donor site scars [6–9, 11, 16–21, 25].

### Primary objectives

This research study aims to establish evidence for tissue support (4–6 mmHg) in healing burn donor sites and to test the use of interim garments made from tubular support bandages for donor site scar management. Specific aims are to investigate whether low-pressure IPG support to donor sites compared with no IPG results in

- Reduction of time to epithelisation
- Reduction of erythema
- Reduction of scar thickness and/or height
- Improvement of overall scar appearance
- Reduction of self-reported pain levels

## Methods/design

### Protocol and registration

The methodology has been documented in a protocol and registered on a publicly available site (Australian New Zealand Clinical Trials Registry [ACTRN] identifier 12610000127000). The methods are summarised here according to the revised Consolidated Standards for Reporting Trials (CONSORT) statement [28].

### Design

The clinical trial has been designed as a single-centre, prospective, single-blind randomised controlled trial.

### Randomisation

Randomisation will be achieved through computerised tabulation with concealed allocation to each participant via a sealed envelope method. Participants will be randomly allocated into one of two groups: a control group that will have no compression to donor sites (the standard existing treatment) or an experimental group that will be provided with compression to donor sites (the comparative treatment).

### Eligibility

All new patients admitted to the Professor Stuart Pegg Adult Burns Centre who require SSG surgery for burn management over less than 15 % of their total body surface area (TBSA) will be screened for eligibility to participate in the study by the principal investigator.

**Table:** 

*Inclusion criteria*	*Exclusion criteria*
Donor site located on one thigh only (measured to be 10 cm above the patella)	Burns to left, right or both thighs
Male or female	Donor site at anatomic location other than the thigh,
Older than 14 years of age (with adult consent) and able to provide informed consent	A psychiatric history documented in the medical chart which could cause a variable outcome (high risk of non-compliance)
	A pre-existing co-morbidity associated with delayed healing
	Arterial disease and/or vascular compromise as previously documented in the medical chart
	A dermatological condition in the region where the donor site needs to be taken
	Allergy to ultrasound gel
	Medications that may impact on wound healing
	Consent not given

If eligibility is established, patients will be approached by the principal investigator and a lay explanation of the study procedures and requirements will be provided. Participants will be included once informed consent has been obtained and documented. Child assent will be documented where appropriate.

### Intervention

The treatment will be IPG objectively measured in millimetres of mercury using the PicoPress® pressure sensor (mediGroup Australia, Melbourne, Australia) [29], and it will be administered post-operatively between days 3 and 5. Participants in the treatment group will be asked to wear the IPG for 23 h/day for a period of 8 weeks. Individual wear time for the IPG will be established through completion of a daily log book for the treatment group. Participants will commence involvement in the study as inpatients on the burn ward and may progress to the outpatient clinic during the study as per normal hospital procedure. Once discharged from the inpatient burn ward, participants will return to the hospital for fortnightly study measurements. Both groups will be reviewed every 2 weeks over the 2-month period to check scar and/or IPG quality (in millimetres of mercury). Surgical intervention, wound care dressings, medication, nutrition, exercise and nursing management of the donor site will be standardized between groups, the sole difference being that one group will have compression therapy for their donor site and the other will not.

The principal investigator will record demographic information, including age, skin type (as assessed using Fitzpatrick skin typing) [30], cause of injury, TBSA burned, depth of injury, surgical procedure undertaken, medication used and time taken to heal (including number of dressing changes and types of dressings used). Smoking status (which may impact on wound healing) will also be recorded. The donor will be considered healed when there is no raw, open or oozing epithelium. A second researcher blinded to the intervention will complete all outcome measures.

### Measurement tools

In this study, the commercially available, evidence-based measurement tools described below will be used. These outcome measures will be done every 2 weeks for a period of 2 months.

#### Scar depth assessment

The DermaScan C (Cortex Technology, Hadsund, Denmark) is a high-frequency, high-resolution ultrasound scanner which objectively captures and reproduces skin thickness. It can clearly discriminate between hypertrophic scar, normal scar and normal skin and is evidenced as having high inter-rater/intra-rater reliability [31, 32].

#### Erythema assessment

The DSM II ColorMeter (Cortex Technology) is a self-contained, battery-operated device which provides objective measurements of erythema and melanin based on the light absorption characteristics of human skin [33, 34].

#### Garment pressure assessment in mmHg

The PicoPress® pressure sensor has been clinically tested for validity and reproducibility in objectively measuring the pressure in millimetres of mercury of compression garments. It has been shown to be an adequate and reliable instrument for use [29, 35].

#### Subjective scar assessment

The Patient Observer Scar Assessment Scale has been shown to consistently demonstrate valid and reliable measures of scar quality [36]. It is a simple and cost-effective assessment that reports the patient’s opinions of the scar, including pain, itch, colour, stiffness, thickness, irregularity and overall appearance, and the observer assessment of pigmentation, thickness, relief, pliability, surace area, height, vascularity and overall appearance of the scar [36–39].

#### Conclusion of assessments

For participants in the IPG group, the principal researcher will measure the amount of pressure in each garment and issue new interim garments if needed, thereby ensuring that pressure remains constant. This concludes the assessment and treatment regime. The participant will undergo the same regime at each subsequent appointment for the period of 8 weeks. At this point, they will be released from the study.

### Standardization

Considerations to standardize each measurement will include the following:

- Measurement of the donor site with the garment removed for a specific length of time (5 minutes)
- Temperature-controlled room
- Position of donor anatomy dermatome location L3–L5 anterolateral thigh
- A template on the donor site to identify the area to be measured
- Time of day when measured

### Sample size

Previous studies using the DermaScan C have shown an effect size of 2.35 (1.69–3.01) between skin-thickness measurements of donor sites and scar sites [22]. For a minimum clinical difference of 0.5 mm between groups of less than 0.05 and a power of 0.9, 22 subjects per group would be required [22, 40] (assuming a standard deviation of 0.5 mm for wound thickness).

On the basis of data obtained from January 2013 to May 2015 (where patients admitted to the RBWH Professor Stuart Pegg Adult Burns Centre are represented as males = 593 admissions and females = 237 admissions), the ratio is expected to be 3:1. This ratio should accurately reflect the distribution of burns within the general community.

### Proposed data analysis

Groups will be compared at baseline regarding the important prognostic indications: donor site location, depth, size, age, skin type and time since graft. Data will be entered into IBM SPSS 22.0 for Windows software files (IBM, Armonk, NY, USA) and tested for normality of distribution. Descriptive statistics for all variables will be computed. If parametric statistics are able to be used, a two-way analysis of variance for group × time interaction will be performed to compare skin thickness between normal, donor and scar sites with a planned least significant difference post hoc analysis. Significance will be set at *p* < 0.05. If raw data are not normally distributed, an equivalent non-parametric analysis will be used [40].

### Ethics, informed consent and safety

This study has received ethical approval from the RBWH HREC (approval number HREC/09/QRBW/327) and the UQ MREC (approval number MREC2010000356). The trial has been registered on a publicly available site (Australian New Zealand Clinical Trials Registry [ACTRN] identifier: 12610000127000). The study will be monitored by the data and safety monitoring board, and any adverse or unforeseen events will be reported (as soon as or before they arise) to the appropriate committee for review (RBWH HREC/UQ MREC).

## Discussion

Donor sites, regardless of their size, are consistently recognised as a potential source of concern. Interventions that assist to accelerate healing and reduce scar formation would considerably improve post-operative management of donor sites [14]. This study aims to provide a better understanding of the effects of IPG (4–6 mmHg) on donor site healing, which is currently poorly represented in the literature.

If IPG is shown to improve donor site scar maturation and healing, it is anticipated that this outcome may assist in improving patient comfort, reduce the incidence of abnormal scarring and reduce the associated psychological stress of this additional wound [13]. Furthermore, such an outcome may allow the healed donor site to be re-harvested more quickly for SSG in burn patients with large TBSA burns and limited donor site availability. If IPG (4–6 mmHg) is shown to make no difference in donor scar healing, this outcome will provide valuable information with regards to already-expanding budget requirements for the future of burns scar management.

### Consent

Written informed consent was obtained from the patient for publication of the images in this article. A copy of the written consent form is held by the authors and is available for review by the Editor-in-Chief of this journal.

### Trial status

Eight participants have been recruited to date for a pilot study. A total of 44 participants (22 for each group) is required.

## Abbreviations

IPG: interim pressure garment
PGT: pressure garment therapy
RBWH HREC: Royal Brisbane and Women’s Hospital Human Research Ethics Committee
SSG: split-thickness skin graft
TBSA: total body surface area
UQ MREC: University of Queensland Medical Research Ethics Committee
